# Telehealth-Plus-mHealth Intervention for Young Adults With Cannabis Use Disorder: Protocol for a Pilot Randomized Controlled Trial

**DOI:** 10.2196/105908

**Published:** 2026-08-20

**Authors:** Lydia A Shrier, Madison M O'Connell, Avery Palmer, Sarah Parker, Joe Kossowsky, Sabra L Katz-Wise, Pamela J Burke, Carly E Milliren, Sion Kim Harris

**Affiliations:** 1 Department of Pediatrics, Harvard Medical School, Boston, MA, United States; 2 Division of Adolescent/Young Adult Medicine, Boston Children's Hospital, 300 Longwood Avenue, Boston, MA, 02115, United States, 1 617-355-8306, 1 617-730-0004; 3 Department of Anesthesiology, Critical Care, and Pain Medicine, Boston Children's Hospital, Boston, MA, United States; 4 Department of Anesthesia, Harvard Medical School, Boston, MA, United States; 5 Division of Sleep Medicine, Harvard Medical School, Boston, MA, United States; 6 Department of Social and Behavioral Sciences, Harvard T.H. Chan School of Public Health, Boston, MA, United States; 7 Biostatistics and Research Design Center, Boston Children's Hospital, Boston, MA, United States; 8 Department of Psychiatry and Behavioral Sciences, Boston Children's Hospital, Boston, MA, United States

**Keywords:** cannabis-related disorder, behavior-change intervention, telehealth, mobile health, mHealth, young adults, motivational interviewing, ecological momentary assessment

## Abstract

**Background:**

More than 5 million young adults in the United States meet criteria for cannabis use disorder (CUD), placing them at risk for adverse physical, psychiatric, and social outcomes. Most individuals with CUD do not receive treatment due to numerous barriers, including motivation, stigma, limited availability and accessibility of developmentally appropriate services, and competing demands for time and finances. A novel digital health intervention, Momentary Self-Monitoring and Feedback + Motivational Enhancement Therapy—Virtual (MOMENT-V), was developed and combines telehealth motivational enhancement therapy with mobile health ecological momentary intervention (EMI) to provide a fully remote brief intervention for CUD among young adults.

**Objective:**

We describe the protocol for a parallel, 2-arm, pilot randomized controlled trial of MOMENT-V vs enhanced usual care (EUC) to evaluate intervention and trial feasibility in young adults with CUD recruited from primary care clinics.

**Methods:**

We are inviting primary care patients, aged 18 to 26 years, to self-screen through clinic posters with QR codes, invitations from clinicians, and patient portal messages. Eligible patients (eg, Cannabis Use Disorder Identification Test-Revised score ≥12, cannabis use ≥3 days/week, and smartphone ownership; N=60) will be randomly assigned 1:1 to receive MOMENT-V or EUC. MOMENT-V will include 2 weekly sessions of motivational enhancement therapy via telehealth, then 2 weeks of mobile health EMI—mobile self-monitoring with messages following reports of personal triggers for use. EUC will include a brief meeting with a counselor to review cannabis use problems and provide substance use and mental health resources. Participants will be assessed via surveys and a timeline followback calendar interview at baseline, 3 weeks, 3 months, and 6 months, and by an interview at 6 months. Primary outcomes will include completion, EMI engagement, and acceptability as measures of intervention feasibility, and screening, eligibility, enrollment, and retention rates as measures of trial feasibility. Primary outcomes will be evaluated against a priori benchmarks. Secondary outcomes will include counselor adherence to motivational interviewing principles, therapeutic alliance, duration of study activities, barriers, facilitators, CUD symptoms, and amount of delta-9-tetrahydrocannabinol used in standard units for motivation to change cannabis use, psychological distress, cognitive function, and quality of life. We will also examine cannabis use frequency and problems resulting from cannabis use. For the quantitative analyses, we will use descriptive statistics, logistic regression, and generalized linear mixed effects modeling. For the qualitative analyses, we will use immersion/crystallization, template organizing style, and thematic analysis.

**Results:**

Recruitment began in August 2025 and is anticipated to end in January 2027. Data collection is projected to be completed in July 2027. We plan to submit a manuscript describing the main outcomes in early 2028.

**Conclusions:**

This study will provide data regarding the MOMENT-V digital health intervention and trial feasibility in anticipation of a fully powered efficacy trial in young adults with CUD who are being seen in primary care.

## Introduction

### Cannabis Use Disorder in Young Adults: High Prevalence, Low Treatment Receipt

Frequent cannabis use has increased in the last 20 years to reach historic levels among young adults in the United States [1], with rates of daily use in this age group as high as 19%‐21% in states that have legalized recreational cannabis use [2]. Approximately 1 in 3 young people who use cannabis regularly (weekly or daily) meet criteria for cannabis use disorder (CUD) [3], an estimated 5.5 million young adults in 2024 [4]. Concurrent with the increase in frequent cannabis use, there has been a >3-fold increase in the potency of cannabis products [5], a substantial increase in vaping of high-potency cannabis oils among young adults [1], and a rise in emergency department visits involving cannabis, particularly for adolescents and young adults [6,7]. Heavy cannabis exposure during brain development (through the 20s) [8] is associated with increased risk of CUD, other substance use disorders, psychosis, and other psychiatric disorders, as well as decreased educational and occupational success [9-12]. Furthermore, cannabis use is associated with adverse cardiovascular and pulmonary effects, and CUD resulting in hospital-based care has been linked to premature death [13].

Increases in the legalization of recreational cannabis and declines in the perceived risk of harm from cannabis have heralded not only increases in cannabis use, but also decreases in the perceived need for and use of CUD treatment among young adults [14-16]. Consequently, despite the substantial need, fewer than 3% of young adults with past-year CUD receive outpatient treatment [14]. Specific barriers to young adults seeking treatment include not feeling worthy of support, fearing stigma, having financial concerns, and encountering logistical or structural issues, including limited availability and accessibility of developmentally appropriate services [17,18]. There is an urgent need to develop, evaluate, disseminate, and implement interventions for young adults with CUD that address barriers to and reduce disparities in receipt of effective treatment [16,19].

### Young Adults and Brief Motivational Interventions for CUD

Motivational interventions – including motivational enhancement therapy (MET), which incorporates motivational interviewing (MI) with personal feedback [20] – are at the forefront of brief substance use treatment [21-24]. Individuals aged 18 to 26 years are in the developmental period between adolescence and adulthood, when they are focusing on themselves and exploring their identity, a time ripe for reflection [25]. During this time, young adults may both be engaging in health-risk behaviors, such as frequent cannabis use, and be open to change to better align their behaviors with their life goals. Consistent with this, baseline motivation to change predicts decreases in cannabis use and associated problems [26-30]. Thus, it is particularly important and salient to develop and test motivational interventions for young adults.

Brief interventions (1‐2 sessions) for young adults do reduce CUD symptoms and increase the likelihood of abstinence in the short term [21], but effects are small and not sustained [31-33]. Psychosocial interventions for adults with CUD that have more than 4 sessions and last more than 1 month have better outcomes [34]. Combining a motivational intervention with other approaches may enhance effectiveness [35], but it adds time, expense, and training needs. Novel approaches to brief motivational interventions for young adults with CUD are required to address the high unmet need, age-specific barriers and facilitators, and challenges to achieving long-term effectiveness.

### CUD Brief Intervention for Young Adults in Primary Care

Primary care is a promising setting in which to offer treatment for CUD in young adults. Most young adults have a primary care provider [36] and have had a wellness visit in the past year [37]. SBIRT (Screening, Brief Intervention, and Referral to Treatment) is already recommended as part of routine care to enable early intervention for those with problematic substance use [38,39]. Young adults disclose cannabis use during screening in primary care at rates similar to or higher than those in surveillance surveys [40]. In one large health care system, 31% of primary care patients aged 18 to 29 years reported using cannabis in the past 3 months, of whom 39% were at moderate to high risk of CUD [41]. Using trained interventionists to deliver treatment has facilitated sustained SBIRT in primary care [42,43]. However, for CUD interventions for large numbers of affected young adult patients to be implemented at scale, challenges with staffing, time, cost, and space in primary care must be overcome [44].

### Digital Health Interventions for Primary Care–Based Treatment of Young Adults With CUD

Digital health interventions, including telehealth, websites, and mobile health (mHealth) apps, offer means of addressing implementation challenges in primary care while leveraging the trust in familiar clinicians, staff, and systems—a cornerstone of young adult care-seeking and disclosure behaviors [45]—to support engagement with treatment. In a meta-analysis, at the 3-month follow-up, digital health interventions for young adults reduced cannabis use by almost 1 week in the previous month, compared with control conditions [46]. Telehealth has been widely adopted in primary care, with more than 70% of physicians working in practices that offer videoconferencing with patients [47].

Because telehealth allows open and nonjudgmental live communication, expressions of acceptance and compassion, and the building of rapport between counselor and client, telehealth may be better suited to motivational interventions than computerized or online interventions [48]. Adding mHealth interventions (eg, SMS text messaging, momentary assessment, and intervention) to face-to-face counseling can improve psychiatric treatment outcomes [49], suggesting that a telehealth-plus-mHealth intervention could be an efficient and effective approach to CUD treatment for young adults in primary care.

### The Momentary Self-Monitoring and Feedback + Motivational Enhancement Therapy—Virtual Intervention

**Figure 1.**
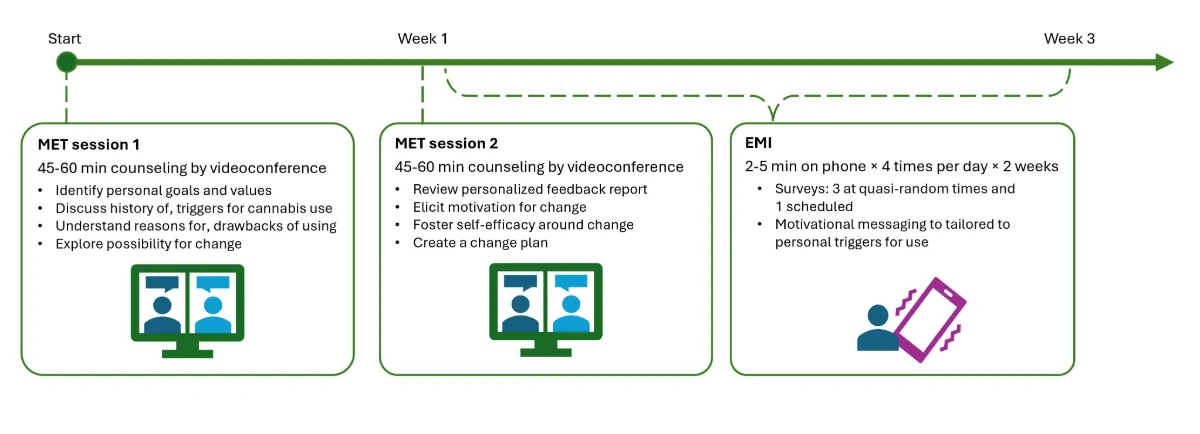
The Momentary Self-Monitoring and Feedback + Motivational Enhancement Therapy—Virtual (MOMENT-V) intervention. EMI: ecological momentary intervention; MET: motivational enhancement therapy.

Based on our prior observational and intervention research [50-52], we developed a telehealth-plus-mHealth intervention for young adults with CUD to be delivered in primary care. The Momentary Self-Monitoring and Feedback + Motivational Enhancement Therapy—Virtual (MOMENT-V) intervention includes 2 MET sessions delivered by telehealth, followed by 2 weeks of ecological momentary intervention (EMI; a brief assessment and intervention in real time and in real life) (Figure 1) [53]. The MET involves 2 manualized 45- to 60-minute sessions, 1 week apart, with a counselor who is a clinician trained in behavior change counseling (eg, a psychologist or nurse practitioner). Sessions include engaging hands-on and cognitive activities. In the first session (~45‐60 min), the counselor establishes rapport; elicits personal goals and values; discusses the young adult’s cannabis history, behaviors, reasons, and expectancies; briefly discusses other substance use; develops a discrepancy between use and the young adult’s values and goals; and explores the possibility of change. During this session, the young adult identifies their triggers for cannabis use from lists of affective states and social contexts, then selects their top 3 triggers. In the second session (~30‐45 min), the counselor provides personalized feedback; evokes motivation for changing use; conducts a decisional balance exercise; assesses the importance of and readiness for change; explores and enhances self-efficacy for change; helps the young adult develop a change plan (“My Journey”); reviews withdrawal symptoms; and assesses confidence in their ability to change.

Following the second MET session, participants receive electronic notifications to respond to 3 momentary surveys and 1 daily survey each day for 14 days. The momentary surveys are prompted at quasi-random times in the morning, afternoon, and evening to assess momentary experience. The momentary surveys query social and emotional contexts associated with the desire to use cannabis and with subsequent use, reasons for use, use of cannabis and other substances, avoidance efforts, and coping strategies. When participants indicate exposure to a personal top-3 trigger context in a momentary survey, they immediately receive a message supporting self-efficacy, evoking the behavior change plan they developed in MET, and prompting them to consider their personal coping strategies, drawing on research indicating the effectiveness of cognitive behavioral therapy combined with MET [54-56]. Participants also receive messages after reporting desire for cannabis, use, and efforts to avoid use. One survey is prompted at a time of the participant’s choosing to permit a daily summary of and reflection on use behaviors, social and emotional contexts, craving, and withdrawal symptoms. Each survey takes 2 to 3 minutes, depending on reported use and the number of messages.

In our previously developed intervention, which comprised in-person MET and an EMI, nearly all participants who were exposed to the MET rated it as excellent or good and reported changes in their cannabis use motivation and behavior related to cannabis use after counseling [51]. We applied our framework to adapt in-person counseling for virtual delivery, updated the counseling language, and created new materials to enhance the sessions. Additionally, we created a website to host intervention materials and resources for participants. We also developed new EMI messaging content and format to improve integration with the MET, expanded the message bank, and implemented a programming algorithm to minimize repetition over the 2-week EMI.

Following human-centered design principles [57], we approached the challenges of CUD treatment in primary care generally and of MOMENT-V specifically by centering on the people experiencing the challenges: young people and their clinicians [51]. Accordingly, we worked with youth throughout the adaptation process, including development, testing, review, and revision. Youth were directly involved in decisions regarding activity design, interactivity, functionality, and order, as well as the balance between face time and screen-sharing during the video counseling sessions. We also worked with mental health and medical clinicians in primary care to develop robust safety procedures for the virtual intervention. We then conducted an open pilot study with primary care patients aged 18 to 25 years who reported recreational cannabis use at least 3 times per week (N=14) [51]. All participants completed the 2 MET sessions and at least 1 EMI survey and reported high satisfaction with the intervention. Confidence to change cannabis use was higher postintervention and at 2-month follow-up, compared to baseline. Additionally, cannabis use frequency decreased by the 2-month follow-up.

### Study Objectives

Building on this strong theoretical and empirical foundation, we now seek to determine intervention and trial feasibility in a pilot randomized controlled trial (RCT) of MOMENT-V vs enhanced usual care (EUC) in young adult primary care patients with CUD. For intervention feasibility, the primary outcomes will be completion, EMI engagement, and acceptability; the secondary outcomes will be counselor adherence to MI principles and therapeutic alliance. With an eye on expanding the reach, accessibility, and engagement in substance use research, as well as in interventions for young adults, we will evaluate remote trial feasibility, with primary outcomes of screening, eligibility, enrollment, and retention rates, and a secondary outcome of duration of study activities. In anticipation of a future efficacy trial, we will examine cannabis use frequency and problems from cannabis use, as well as CUD symptoms, amount of delta-9-tetrahydrocannabinol (THC) used, motivation to change use, psychological distress, cognitive function, and quality of life.

## Methods

### Trial Design

The study design is a 2-arm, parallel group, pilot RCT with 1:1 allocation of young adult primary care patients with CUD to MOMENT-V or EUC. Following self-screening indicating eligibility, patients will be scheduled for a remote baseline visit, at which they will be enrolled, randomly assigned to their study condition, and will receive their assigned condition. During remote study visits at baseline, 3 weeks, 3 months, and 6 months, the research assistant will send the participant electronic surveys to complete during the visit and will conduct timeline followback (TLFB) interviews [58]. In addition, at the end of the 6-month visit, the research assistant will conduct a semistructured feedback interview. The study includes an exploration of the use of oral fluid testing to evaluate change in cannabis use in clinical trials. The oral fluid testing protocol will be described in a future publication.

### Participants

Participants will be aged 18 to 26 years, be primary care patients at a recruitment clinic, and have a CUD Identification Test-Revised (CUDIT-R) score ≥12 or more. The CUDIT-R is a self-report measure assessing problematic cannabis use during the past 6 months, with 7 items, each rated on a 5-point Likert-type scale from 0 to 4, and 1 item rated 0, 2, or 4; item scores are summed, and scores of ≥12 are 91% sensitive and 90% specific for CUD [59]. Participants will also meet these additional inclusion criteria: recreational cannabis use on 3 or more days per week, on average, in the past 30 days; ownership of a smartphone; ability to read and speak English; and availability for the 6-month study duration. Participants will not meet any of the following exclusion criteria: a medical or psychiatric condition that, in the opinion of the study principal investigator, would prevent safe participation in the study; inability or unwillingness to provide contact information; written certification from a physician for marijuana for medical use; currently receiving specialized counseling or treatment services for a substance use problem; or participation in prior MOMENT or MOMENT-V research. As in our prior research in young adults with CUD, we will also exclude individuals who are pregnant or parenting owing to safety and social concerns or potential disclosures that cannot be adequately addressed in our brief, remotely delivered intervention and EUC conditions. Pregnancy and parenting status will be self-reported on the electronic eligibility survey.

Participants in both conditions will continue to receive standard clinical care, which could include regular follow-up visits or referrals to behavioral health or substance use treatment, as determined by a clinician caring for them. Receipt of care outside the study will be assessed on each follow-up survey. Participants will be remunerated up to US $260 in e-gift cards, prorated based on completion of study activities. Payment will be based solely on completion of study activities and will not be contingent on cannabis use, abstinence, or any other outcome. Participation in the study is voluntary, and participants may choose to leave the study at any time.

Patients at the recruitment clinics aged 18 to 26 years who have a history of problematic cannabis use but do not currently have a CUDIT-R score of 12 or more (and are therefore ineligible to enroll in the study) may be invited to participate in the study’s Advisory Board. The Advisory Board will meet 1‐2 times a year for approximately 2 years during participant-related procedures (ie, recruitment, enrollment, intervention delivery, and assessment) and data analysis. The Advisory Board will be asked to provide feedback on study materials, procedures, findings, and dissemination plans.

### Setting and Recruitment

Patients will be recruited from clinics in eastern Massachusetts that provide primary care to young adults. The initial recruitment sites will be 2 clinics affiliated with a pediatric teaching hospital. Additional sites (eg, Boston-area community health centers) will be added as needed to meet enrollment goals. In Massachusetts, recreational cannabis use is legal for individuals aged 21 years and older [60].

Patients will be invited to self-screen for the study through flyers with a QR code displayed in clinics, clinician invitations, and research assistant invitations (eg, patient portal messages). The QR code will link to a self-administered screening survey assessing eligibility and collecting contact information. The survey will also collect the name of the patient’s primary care clinician to contact if the patient reports a safety concern. The survey will include information about how to reach a clinician at the clinic if questions or concerns arise while completing the survey.

### Enrollment and Randomization

The initial study visit will occur via a video call. Following enrollment, the participant will self-administer the baseline survey on demographic characteristics and cannabis use-related history, symptoms, and problems. After the participant has completed the survey, the research assistant will conduct a substance use TLFB calendar interview for the past 30 days. The research assistant will then initiate the electronic randomization module and view the participant’s assigned condition. The research assistant will then invite the study counselor to join the video call.

### Conditions

#### MOMENT-V Intervention

At the baseline video visit, the participant will meet with a study counselor for the first MET session. One week later, the participant will have a video visit with the study counselor for the second MET session. At the end of the second MET session, the research assistant will orient the participant to the EMI, explaining that for 14 days the participant will receive 3 notifications per day at quasi-random times in the morning, afternoon, and evening (“momentary surveys”) and 1 notification per day at a time of their choosing (“daily diary”). If the participant does not have a passcode or password on their smartphone, they will be asked to add one. The research assistant will ask the participant to indicate their preferred time for the daily diary. The EMI will start the day after the visit and continue for 2 weeks. After the first 48 to 72 hours, the research assistant will send a message by text or email, according to the participant’s preference, to check in. For response rates below 70%, the message will advise the participant to seek technical help and remind the participant to respond.

#### EUC

The study counselor will meet with participants assigned to EUC to review their problems with cannabis use (items they endorsed on the CUDIT-R), advise them to talk with their primary care clinician about their cannabis use, and share local and national substance use and other behavioral health resources. Participants in the EUC condition will receive a link to the resource list and will be able to access the list through the study website. We selected an EUC comparator in accordance with expert recommendations to use comparators that align with the primary study purpose (ie, to prepare for the fully powered RCT), are clinically relevant, and, in early-phase research, are not excessively formidable [61]. We chose to enhance standard care to represent best practice—once we have identified CUD, we want to ensure that participants who are not assigned to the intervention receive information about treatment resources from a clinician.

#### Training and Fidelity Monitoring

The study counselors will be clinicians (eg, psychologists and nurse practitioners) with training in MI for behavior change. They will receive training on the MOMENT-V intervention using a detailed intervention manual and slides for intervention delivery, including didactic instruction from the principal investigator, self-study, and practice with mock participants (with a total training duration of at least 8 h). All MET sessions with participants will be videorecorded and transcribed. After each MET session, study counselors will complete a session-specific checklist documenting adherence to the MET manual and to MI principles [62]. Trained research staff will also rate the recorded MET sessions for protocol adherence and counselors’ competency in using MI. Each counselor’s first pair of MET sessions and 1 additional randomly selected pair of sessions will be rated (with 3 counselors, n=6 session 1 and n=6 for session 2). Based on previous research, the suggested frequency of monitoring competency during the first 6 months after training is 3 to 4 times (ie, every ~6‐8 wk) [63]. These coaching meetings will enable the principal investigator to provide ongoing feedback. In our previous research, we demonstrated that the counselors maintained high fidelity to MI and to the manual [51]. After the initial 6 months, assessments may be conducted less often (ie, every ~12 wk) as long as counselors continue to demonstrate competency. If there is evidence of declining fidelity to the MET, the principal investigator will provide additional coaching and, if necessary, retraining.

### Outcomes

We will assess the study outcomes using self-report surveys, TLFB calendar interviews, semistructured feedback interviews, and study tracking logs at baseline, postintervention, and 3-week, 3-month, and 6-month follow-ups.

#### Intervention Feasibility Outcomes

The primary outcomes for intervention feasibility include completion, EMI engagement, and acceptability. For intervention completion, we will calculate the percentage of participants assigned to MOMENT-V who complete both MET sessions and any EMI surveys (benchmark: ≥80%). For each MOMENT-V participant, we will determine EMI engagement by calculating the percentage of days (out of a possible 14 d) on which the participant responds to at least 1 EMI survey (benchmark: median ≥80%) [64]. For acceptability, at 6 months we will examine participant satisfaction measured by the Client Satisfaction Questionnaire–8 [65]; each of the 8 items is scored from 1 to 4, and the total score is determined from the sum of the item scores (range of possible total scores, 8‐32), with higher scores indicating greater satisfaction (benchmark: ≥80% of participants with a scale score ≥24, indicating a mean item score of ≥3 out of 4). We will also examine the acceptability of the MET sessions and EMI components using previously-developed scale measures administered following each activity (scales of 11‐12 items with 5-point Likert-type response options from 1=“Strongly Disagree” to 5=“Strongly Agree”); higher scores indicate greater acceptability (benchmark for each scale: ≥80% of participants with a mean item score ≥4 out of 5).

The secondary outcomes for intervention feasibility include the counselor’s adherence to MI principles during MET delivery and the therapeutic alliance. We will calculate session-specific adherence scores on scales that use 7‐9 items adapted from the Behavior Change Counseling Index [62] with 5-point Likert-type response options from 1 (“Strongly Disagree”) to 5 (“Strongly Agree”). Adherence scales will be administered after each MET session; higher scores indicate better counselor adherence to MI principles during the session (benchmark for each scale: ≥80% of participants with a mean item score ≥4 out of 5). After the second MET session, we will measure therapeutic alliance using the Working Alliance Inventory–Short Revised (WAI-SR) [66], which includes 12 items with 5-point Likert-type responses and has demonstrated good psychometric properties. The WAI-SR assesses three aspects of therapeutic alliance (each with a 4-item subscale): agreement on therapy tasks, agreement on therapy goals, and development of an affective bond between the individual and the therapist. We will examine total and subscale scores (benchmark: ≥80% of participants with a total score ≥48 indicating a mean item score ≥4). Both participants and counselors will complete the WAI-SR to permit testing for agreement.

The semistructured interview at 6 months will further assess intervention feasibility and acceptability with questions on facilitators, barriers, preferences, and recommendations related to the intervention (for participants assigned to MOMENT-V) and to treatment in general (for participants assigned to EUC).

#### Trial Feasibility Outcomes

The primary outcomes for trial feasibility include the rates of screening, eligibility, enrollment, and retention. The secondary outcomes are the durations of the study activities, measured in minutes for each activity. The 6-month semistructured interview will include questions on facilitators, barriers, preferences, and recommendations related to completing study activities.

#### Preliminary Efficacy Outcomes

To prepare for a fully powered trial, we will examine recommended primary and secondary outcomes for the efficacy of the intervention [67-69]. The primary preliminary efficacy outcomes are the number of days of cannabis use, the number of times of cannabis use, and the negative consequences of cannabis use. Days of cannabis use are consistently associated with clinical outcomes, and evidence to date supports the use of this measure to determine cannabis reduction in clinical trials [70]. The number of self-reported days of cannabis use in the past 30 days will be assessed via a TLFB calendar at baseline, 3 months, and 6 months; cannabis use days in the past 14 days will be assessed via TLFB at 3 weeks. For each day with cannabis use, participants will be asked to estimate the time(s) of cannabis use and report their certainty about the time(s) to capture information about changes in total cannabis exposure [70]. The number of self-reported negative consequences of cannabis use in the past 3 months will be measured on the 19-item (yes or no) Marijuana Problems Scale at baseline, 3 months, and 6 months [71].

The secondary preliminary efficacy outcomes will include number of CUD symptoms, amount of THC used, motivation to change cannabis use, psychological distress, cognitive function, and quality of life. Number of CUD symptoms (per the *Diagnostic and Statistical Manual of Mental Disorders*, Fifth Edition [DSM-5]) [72] will be measured by the Composite International Diagnostic Interview–Substance Abuse Module at baseline, 3 months, and 6 months [73]. We consider symptom count to be a secondary outcome because its validity to assess change at 3 and 6 months postintervention in young adults with CUD has not been established. Because participants may change their cannabis use by changing THC potency, we will estimate the amount of THC used from data on the forms and amounts of cannabis collected during the 3-month and 6-month TLFB assessments per international expert consensus recommendations (iCann Toolkit) [69,74]. Number of standard THC units (1 unit=5 mg THC) [75] will be derived from the number of familiar units (juniors, vapes, gummies, etc) multiplied by the amount of cannabis per unit multiplied by the estimated potency [76]. If it is not possible to calculate standard THC units (eg, because complete data are not available), then we will consider the number of familiar units to measure the amount of THC used. We will measure motivation to change at all time points with 0‐10 rulers for readiness, importance, and confidence not to use cannabis [20,53,77]. Functioning will be assessed at baseline, 3 months, and 6 months using validated measures: psychological distress on the Kessler Psychological Scale-6 [78], cognitive function on the PROMIS Cognitive Function v.2.0—Short Form 6a [79], and quality of life on the PROMIS Global Health v1.2 [80].

### Harms

This study confers no more than minimal risk. The study topic could make participants feel uncomfortable or upset, in which case they may skip questions or decide not to continue. Participants could be prompted to answer EMI surveys in situations that may be inconvenient or dangerous, such as during class or while driving; they will be encouraged to use their best judgment about when to complete them. There is a risk of breaches of privacy and confidentiality; participants will be advised to complete study activities in private places where they will not be seen or overheard, and they will be informed that their data will be stored securely. As part of the informed consent process, participants are also advised that the study team will notify their primary care clinician of their enrollment in the study and that, if they meet a clinically informed safety threshold, the clinician will be notified without revealing the participant’s specific responses. Safety thresholds include any of the following self-reported behaviors over the past 30 days: recent binge drinking or heavy alcohol use (male individuals: >5 drinks within 2 h or on any day, or >15 drinks per week; female individuals: >4 drinks within 2 h or on any day or >8 drinks per week), illicit substance use (eg, anything besides alcohol or cannabis to get high), or operation of a motor vehicle within 3 hours of consuming a psychoactive substance. The study team will request clinician confirmation that the notification was received and will follow up as needed. Additionally, confidentiality will be breached in the unlikely event that the participant spontaneously discloses a serious and imminent risk to their safety (eg, suicidality), in which case they will be directly connected with an on-call clinician for standard clinical care. Whether and how the clinician addresses the safety concern with their participating patient will be at the clinician’s discretion and is outside the scope of the study team.

### Ethical Considerations

This paper follows the CONSORT (Consolidated Standards of Reporting Trials) and SPIRIT (Standard Protocol Items: Recommendations for Interventional Trials; Checklist 1) reporting guidelines for randomized trials [81], as well as the guidelines for web-based and mHealth interventions [82]. The study was approved by the hospital Institutional Review Board (IRB-P00049240) and registered on ClinicalTrials.gov (NCT06661031) in October 2024. Protocol amendments will undergo Institutional Review Board approval and will be reported to the National Institute on Drug Abuse as appropriate. The research assistant will facilitate an electronic informed consent process with comprehension checks about study activities, risks, benefits, and confidentiality. Participants will be remunerated up to US $260 in e-gift cards, prorated based on completion of study activities. Payment will be based solely on completion of study activities and will not be contingent on cannabis use, abstinence, or any other outcome. Participation in the study is voluntary, and participants may choose to leave the study at any time. Advisory board members will be offered a US $50 e-gift card per meeting.

### Sample Size

For this pilot feasibility trial, we will enroll 60 patients, whom we will randomize 1:1 to MOMENT-V vs EUC. As our goal is feasibility testing, a sample size calculation is not required. A sample of 60 participants is large enough to describe feasibility constructs. Specifically, for a key measure of trial feasibility, retention, we will be able to estimate a trial retention rate at 3-month follow-up of 80% in a sample of 60 to within a 95% CI of ±10%, that is, 70%-90%, calculated as 1.96 × the square root of [p×(1 p)/n], where p is the percentage retention that we expect to see and n is the intended sample size. Retention efforts will include bimonthly participation reminders, a birthday text, and respectful remuneration.

### Randomization

At the first visit, after completing the baseline assessments, participants will be randomly assigned 1:1 to MOMENT-V vs EUC. Treatment allocation is concealed using a computer-generated urn randomization list via the REDCap (Vanderbilt University) randomization module, which is accessible only to the study biostatistician. Randomization will balance groups by participant factors that may influence study outcomes. Specifically, assignment will be stratified by sex (male vs not male), race (Black or African American vs not Black or African American), cannabis use frequency (daily vs not daily), and recruitment site. Because of the urn randomization algorithm, future assignments cannot be predicted from previous allocations. In addition, study personnel responsible for participant enrollment do not have access to future assignments, preventing prediction of upcoming allocations. The condition will not be masked to participants, study counselors, or research assistants. The condition will be masked to the biostatistician via a numeric binary code.

### Data Collection, Management, and Monitoring

All self-administered surveys will be hosted in REDCap, a HIPAA (Health Insurance Portability and Accountability Act)-compliant platform [83,84]. Validation techniques will be programmed (eg, branching logic, valid numeric ranges, and so on). We will operate under a National Institutes of Health (NIH) Certificate of Confidentiality and use methods to further protect confidentiality and privacy, including assigning a unique numeric study ID to each participant in lieu of storing survey responses with personal information. Data will be exported and stored in a secure, restricted-access location on the hospital’s file network.

The principal investigator will oversee implementation of the study’s data and safety monitoring plan to ensure data integrity and security. Because this is a feasibility trial, there are no plans for interim analyses or stopping rules.

### Data Analysis

#### Quantitative Data

We will examine sociodemographic characteristics and baseline measures to verify that randomization resulted in groups with similar characteristics. We will also use the 2-tailed *t* test (or Wilcoxon-Mann-Whitney test, if data are skewed) and chi-square test (or Fisher exact test) to evaluate equivalence at baseline of the MOMENT-V and EUC groups. We will compute descriptive statistics for baseline sociodemographic characteristics, cannabis use, and related factors.

For the intervention feasibility outcomes, we will calculate the percentage of participants completing the intervention at 6 months and compare it to the benchmark using a chi-square test or Fisher exact test, depending on the data distribution. We will examine sociodemographic and cannabis use determinants of intervention completion using logistic regression models. We will calculate total scores on the acceptability scales and compare them to the benchmarks using a 2-tailed *t* test (or its nonparametric equivalent, the Wilcoxon-Mann-Whitney test, if the data are skewed). Among participants randomized to MOMENT-V, we will summarize each participant’s percentage of days with app engagement using descriptive statistics and compare the percentage of participants completing at least 1 survey per day during the 14-day EMI period to the benchmark. We will examine factors associated with EMI engagement, such as sociodemographic factors and cannabis use characteristics, using multivariable linear or logistic regression. We will use descriptive statistics to summarize ratings of participants’ reports of MET counselor adherence to MI principles and of therapeutic alliance on the WAI-SF, and we will compare the ratings against the benchmarks using a chi-square test (or Fisher exact test).

For the trial feasibility outcomes, we will calculate proportions and 95% CIs for screening, eligibility, enrollment, and retention, and compare them against the benchmarks using chi-square tests. We will also examine the duration of each study activity using descriptive statistics (eg, mean and SD or median and IQR, depending on the distribution and skewness).

We will preliminarily examine the efficacy of MOMENT-V on 30-day cannabis use frequency and on past 3-month negative consequences or problems with cannabis use at 3 and 6 months. To control for potential confounders identified in bivariate analyses of group differences at baseline, we will regress follow-up outcomes on treatment status, adjusting for baseline measures in intention-to-treat analyses. We will conduct analyses using generalized linear mixed-effects models. Models will include the study participant as a random effect and fixed effects for the study arm and potential confounders and covariates such as sociodemographic factors. We will conduct both intention-to-treat and per-protocol analyses to evaluate the robustness of the findings.

#### Qualitative Data

We will examine the domains addressed in the 6-month interview via immersion/crystallization [85], template organizing style [86], and thematic analysis [87] approaches. First, we will use immersion/crystallization to familiarize ourselves with the data by reading a subset of transcripts and creating memos about potential codes and themes [85]. Then a codebook will be developed using a template organizing style with inductive and deductive codes and tested on the same subset of transcripts used for immersion/crystallization [86]. The codebook will be used to code the full set of interview transcripts using the online mixed methods program Dedoose (SocioCultural Research Consultants, LLC). After completing initial coding of all transcripts, we will apply a “code cleaning” process to determine if each code captures a distinct idea, during which some codes will be combined and others will be split into new codes. Finally, themes will be developed using a thematic analysis approach [87], in which data will be consolidated into major themes and subthemes by creating groups of codes that are thematically related. Coding discrepancies and thematic disagreements will be resolved through ongoing discussion and consensus among the coding team. Survey data will be used descriptively to contextualize the interview data.

## Results

We began enrolling participants in August 2025. We anticipate completing enrollment in January 2027 and data collection in July 2027. We will report on the primary outcomes by the first half of 2028, including on ClinicalTrials.gov, where the trial protocol and statistical plan can also be accessed. We will follow NIH data sharing policies to make a deidentified dataset readily available. We intend to present the findings from this study at national scientific meetings and through publications in journals pertaining to adolescent health, substance use, public health, mental and behavioral health, or related fields. In collaboration with the study’s Advisory Board, we will develop and implement plans to disseminate the findings to participants who have elected to receive this information, and to general patient populations, clinicians, and administrators of the recruitment clinics.

## Discussion

### Anticipated Findings

Using mixed methods and a pilot randomized trial design, this study will provide evidence of the feasibility and preliminary efficacy of the MOMENT-V telehealth-plus-EMI intervention to reduce cannabis use among young adults with CUD who are seen in primary care settings. The study will also provide evidence of the feasibility of the remote trial procedures in anticipation of a future fully powered efficacy trial of the MOMENT-V intervention vs EUC.

### Strengths and Limitations

The MOMENT-V intervention has been developed based on a solid conceptual framework, best practices for behavior change counseling, empirical data, and young adult input. This study will use recommended methods to evaluate feasibility; we will collect data on multiple validated and standardized measures and compare the results with a priori benchmarks. Data will be collected electronically, and rigorous procedures to monitor counselor fidelity and ensure data quality will be used. A pilot test of MOMENT-V provided preliminary evidence that young adults with CUD engaged with the intervention and found it helpful [53].

This study may be limited by challenges with recruitment and retention owing to factors outside our control, such as clinic or participant disengagement or competing demands. We will make every effort to support successful recruitment by using inclusive, prominently displayed recruitment materials, developed with input from young adult and community advisors, and by maintaining ongoing communication with clinic staff regarding recruitment and study progress. The research team will meet weekly to review enrollment and promptly identify and address issues. We will mitigate attrition by implementing a detailed informed consent process, offering respectful remuneration, collecting extensive locating information, maintaining frequent contact, and using appealing methods, in addition to conducting all study activities remotely. To further support study engagement, we will invite participant feedback on surveys and in an interview. We will optimize intervention completion and EMI engagement by scheduling MET sessions and report prompts around work/school schedules and sleep hours and by sending text reminders about participation. Participants will not be blinded to their study condition, which may influence their satisfaction ratings, retention, and other outcomes. Because participation is voluntary, the results may not be generalizable to young adults with CUD who are not seeking treatment or not motivated to participate in research.

### Conclusions

The growing public health crisis of CUD [88] in young adults mandates the availability of evidence-based, developmentally appropriate, accessible, affordable, and practical treatment that will be acceptable to patients and clinicians alike. By combining telehealth MET with mHealth EMI, the innovative and scalable MOMENT-V offers a brief fully remote approach for implementation within primary care, addressing many treatment barriers for the vulnerable and underserved population of young adults with CUD. Should the MOMENT-V intervention and procedures prove feasible in this rigorous randomized pilot trial, a large trial will be warranted to evaluate the intervention’s efficacy and advance much-needed research on CUD treatment for young adults.

## Supplementary material

10.2196/105908 Checklist 1 SPIRIT checklist.

10.2196/105908 Peer Review Report 1 Peer-review report from the National Institute on Drug Abuse award R34DA060500-01A1.

## Abbreviations

CONSORT: Consolidated Standards of Reporting Trials
CUD: cannabis use disorder
CUDIT-R: CUD Identification Test-Revised
DSM-5: *Diagnostic and Statistical Manual of Mental Disorders*, Fifth Edition
EMI: ecological momentary intervention
EUC: enhanced usual care
HIPAA: Health Insurance Portability and Accountability Act
MET: motivational enhancement therapy
mHealth: mobile health
MI: motivational interviewing
MOMENT-V: Momentary Self-Monitoring and Feedback + Motivational Enhancement Therapy—Virtual
NIH: National Institutes of Health
RCT: randomized controlled trial
SBIRT: Screening, Brief Intervention, and Referral to Treatment
SPIRIT: Standard Protocol Items: Recommendations for Interventional Trials
THC: tetrahydrocannabinol
TLFB: timeline followback
Working Alliance Inventory–Short Revised: WAI-SR
