# Correction: Telehealth-Plus-mHealth Intervention for Young Adults With Cannabis Use Disorder: Protocol for a Pilot Randomized Controlled Trial

**DOI:** 10.2196/110610

**Published:** 2026-11-02

**Authors:** Lydia A Shrier, Madison M O'Connell, Avery Palmer, Sarah Parker, Joe Kossowsky, Sabra L Katz-Wise, Pamela J Burke, Carly E Milliren, Sion Kim Harris

**Affiliations:** 1 Department of Pediatrics, Harvard Medical School, Boston, MA, United States; 2 Division of Adolescent/Young Adult Medicine, Boston Children's Hospital, 300 Longwood Avenue, Boston, MA, 02115, United States, 1 617-355-8306, 1 617-730-0004; 3 Department of Anesthesiology, Critical Care, and Pain Medicine, Boston Children's Hospital, Boston, MA, United States; 4 Department of Anesthesia, Harvard Medical School, Boston, MA, United States; 5 Division of Sleep Medicine, Harvard Medical School, Boston, MA, United States; 6 Department of Social and Behavioral Sciences, Harvard T.H. Chan School of Public Health, Boston, MA, United States; 7 Biostatistics and Research Design Center, Boston Children's Hospital, Boston, MA, United States; 8 Department of Psychiatry and Behavioral Sciences, Boston Children's Hospital, Boston, MA, United States

In “Telehealth-Plus-mHealth Intervention for Young Adults With Cannabis Use Disorder: Protocol for a Pilot Randomized Controlled Trial” [1], the authors noted one error.

The originally published author order was as follows:

Lydia A Shrier, MD, MPH; Avery Palmer, BA; Sarah Parker, MSN, RN, FNP-BC; Joe Kossowsky, PhD, MMSc; Sabra L Katz-Wise, PhD; Pamela J Burke, PhD, RN, FNP, PNP; Carly E Milliren, MPH; Sion Kim Harris, PhD, MSN, RN, PMHNP-BC, CPH

The correct author order is as follows:

Lydia A Shrier, MD, MPH; Madison M O’Connell, MPH; Avery Palmer, BA; Sarah Parker, MSN, RN, FNP-BC; Joe Kossowsky, PhD, MMSc; Sabra L Katz-Wise, PhD; Pamela J Burke, PhD, RN, FNP, PNP; Carly E Milliren, MPH; Sion Kim Harris, PhD, MSN, RN, PMHNP-BC, CPH

The affiliation for Madison M O’Connell is already present in the paper as affiliation 2 and has been included as such. No further changes were required.

The correction will appear in the online version of the paper on the JMIR Publications website together with the publication of this correction notice. Because this was made after submission to PubMed, PubMed Central, and other full-text repositories, the corrected article has also been resubmitted to those repositories.
