# Supplementary material for: Telehealth-Plus-mHealth Intervention for Young Adults With Cannabis Use Disorder: Protocol for a Pilot Randomized Controlled Trial
Source: JMIR Res Protoc. 2026 Aug 20;15:e105908. doi: 10.2196/105908 (PMC13492189; doi:10.2196/105908)
Supplement: Supplementary file 2 [file resprot-v15-e105908-s002.pdf]

SUMMARY STATEMENT
(Privileged Communication)

PROGRAM CONTACT:
Aidan Hampson
301-827-5925
aidan.hampson@nih.gov

Release Date: **06/27/2024**
Revised Date:

Application Number: **1R34DA060500-01A1**
Formerly: **1R34DA060500-01**

Principal Investigator

SHRIER, LYDIA A.

Applicant Organization: BOSTON CHILDREN'S HOSPITAL

Review Group: **IPTA**
Interventions to Prevent and Treat Addictions Study Section

Meeting Date: **06/13/2024**
Council: **OCT 2024**
Requested Start: **12/01/2024**

Opportunity Number: **PAR-22-183**
PCC: **CC/AJH**

Project Title: **Developing a Telehealth + mHealth Cannabis Use Intervention for Young Adults (MOMENT-V)**
SRG Action: **Impact Score:28**
Next Steps: **Visit https://grants.nih.gov/grants/next_steps.htm**
Human Subjects: 30-Human subjects involved - Certified, no SRG concerns
Animal Subjects: 10-No live vertebrate animals involved for competing appl.
Gender: 1A-Both genders, scientifically acceptable
Minority: 1A-Minorities and non-minorities, scientifically acceptable
Age: 7A-Only Adults, scientifically acceptable

Project Year	Direct Costs Requested	Estimated Total Cost
1	125,000	222,500
2	175,000	311,500
3	150,000	267,000
TOTAL	450,000	801,000

ADMINISTRATIVE BUDGET NOTE: The budget shown is the requested budget and has not been adjusted to reflect any recommendations made by reviewers. If an award is planned, the costs will be calculated by Institute grants management staff based on the recommendations outlined below in the COMMITTEE BUDGET RECOMMENDATIONS section.

1R34DA060500-01A1 SHRIER, LYDIA

RESUME AND SUMMARY OF DISCUSSION: This proposal seeks to use quantitative and qualitative methods to test the feasibility and preliminary efficacy of MOMENT-V, a fully remote telehealth and mHealth intervention in primary care, as a means of facilitating treatment for the vulnerable and underserved population of young adults (YA) with cannabis use disorder (CUD). The project's high significance lies in its ability to advance what is currently known about addressing rising rates of cannabis use disorder in young adults, with a limited number of existing interventions for this age range and multiple barriers to their utilization. The investigative team was considered excellent, with complementary expertise and experience necessary to successfully complete this project, and with access to an excellent and well-resourced research environment. The project was considered innovative, with the fully remote implementation, including bioverification of abstinence through remote oral fluid testing. The panel noted this resubmission to be mostly responsive to the concerns raised in the prior review. The panel was enthusiastic about the many strengths of the application, including the promising preliminary work; intervention delivery via a free mobile app, which contributes to overcoming barriers to access; the YA advisory board; and plans for recruitment through primary care clinics. During the discussion, the panel also noted some weaknesses in the approach, including potentially subjective exclusion criteria; concerns around generalizability, with external counselors not embedded in the primary clinics; and limited clarity regarding how much the current study will improve upon prior pilot work of this group. Concerns were also raised due to insufficient detail provided about the mHealth app, particularly around the user interface and interaction flow of the app. Following the discussion, the panel agreed that the weaknesses identified did not detract from the project's high significance. Findings are expected to have a high to moderately high overall impact on the field.

DESCRIPTION (provided by applicant): Daily cannabis use among young adults aged 18-26 years is at historically high levels and >4.8 million young adults meet criteria for cannabis use disorder (CUD). Heavy cannabis exposure during brain development adversely affects cognition, emotions, risk for psychiatric disorders, and educational and other social outcomes. However, most young adults with CUD do not receive treatment, with disparities by sex and race/ethnicity. Brief motivational interventions may be effective for CUD in young adults, but effects are small and evidence is of low quality. Combining motivational interventions with other approaches may enhance effectiveness, and provision in primary care and use of telehealth may improve reach and reduce disparities. We have developed a novel intervention, MOMENT-V, that combines telehealth motivational enhancement therapy (MET) with mobile health (mHealth) ecological momentary intervention (EMI) to provide a fully remote brief intervention for CUD in young adults seen in primary care, building on our prior research on cannabis use in daily life, a brief intervention with in-person MET plus EMI, adapting in-person counseling for the virtual environment, and brief interventions in primary care. In an open pilot, 100% of young adults were retained, and satisfaction and engagement with MOMENT-V was high. We now propose a pilot randomized controlled trial of MOMENT-V vs. enhanced usual care in 60 young adults aged 18-26 years with CUD recruited from primary care clinics, using mixed methods and with follow-up at 3 weeks, 3 months, and 6 months. The specific aims are to determine 1) intervention feasibility (primary outcomes: intervention completion, EMI engagement, acceptability), 2) trial feasibility (primary outcomes: screening, eligibility, enrollment, retention), and 3) preliminary efficacy (primary outcomes: cannabis use frequency, problems from/negative consequences of cannabis use). Primary outcomes will be evaluated against a priori benchmarks. Secondary outcomes will include MET adherence to motivational interviewing principles, therapeutic alliance, duration of study activities, barriers, facilitators, CUD symptoms, amount of delta-9-tetrahydrocannabinol (THC) used in standard units, motivation to change use, psychological distress, cognitive function, and quality of life. All intervention and trial procedures will be conducted remotely. Additionally, we will explore the feasibility of

videorecorded and live video-observed oral fluid testing for THC and of using these results to assess cannabis reduction in a fully remote trial. This proposal is responsive to PAR-22-183 areas of special interest, including research that uses innovative technologies and Stage I treatment development research testing behavioral interventions within primary care. Results will be used to develop an R01 proposal for a fully powered randomized controlled trial to test the effectiveness of MOMENT-V. Findings from the proposed study will enhance efforts to provide high- quality brief treatment to diverse populations of young adults with CUD and inform research using telehealth, mHealth, remote trial implementation, and oral fluid testing to objectively assess cannabis use reduction.

PUBLIC HEALTH RELEVANCE: Despite rising rates of daily cannabis use in young adults and elevated risk for adverse cognitive, psychiatric, and social outcomes, very few young adults with cannabis use disorder (CUD) receive treatment, with disparities by sex and race/ethnicity. This project will use rigorous quantitative and qualitative methods to test the feasibility and preliminary efficacy of MOMENT-V, a novel, fully-remote telehealth and mHealth intervention in primary care, as a means of facilitating treatment for the vulnerable and underserved population of young adults with CUD.

CRITIQUE 1

Significance: 2
Investigator(s): 1
Innovation: 4
Approach: 3
Environment: 1

Overall Impact: This resubmitted R34 proposal aims to assess initial intervention and trial feasibility and intervention efficacy of a fully remote cannabis use disorder (CUD) treatment delivered through primary care clinics. 60 young adults with CUD will be included and randomized to fully remote MET + EMI or enhanced usual care treatment. The proposal has many strengths, including the timeliness of addressing rising issues of cannabis use disorder in young adults through a high-impact strategy of implementing treatment through primary care in a fully remote design. Further, the intervention design is ideal for overcoming barriers to this population, including using technology young adults are familiar with, using ecological momentary intervention (EMI) to implement the intervention in real life events, reviewing study and intervention design with a young adult advisory board (YAAB), specifically targeting underrepresented populations vulnerable to CUD for treatment, and sharing of treatment manuals and tools with the clinical community. Innovations to the project include its remote and personalized nature and testing of bioverification of abstinence through remote oral fluid testing. On balance, enthusiasm is slightly hampered by the lack of clarity regarding how much the current study will improve upon prior pilot work of this group and the potentially subjective exclusion criteria (i.e., the PI’s opinion regarding comorbidities). Altogether, this proposal is likely to be high impact.

1. Significance:

Strengths

- A fully-remote, primary care-initiated treatment would likely have broad applicability.
- Addressing triggers for cannabis use in momentary, real-life scenarios through EMI is likely to shape the immediate decision making of individuals to use or not use.

- The preliminary pilot data increases likelihood of success of having a significant impact through the thorough consideration of intervention components, including specifically the determination of EMI frequency and content.
- Recruitment of a diverse participant pool and use of a YAAB makes this more impactful.
- Sharing of manuals and tools increase the significance of this proposal as, if they are effective, it will increase access to evidence-based treatment for individuals with CUD.

Weaknesses

- Given the extensive pilot data, it is unclear why a fully-powered RCT is not proposed. While there are changes (improvements) to the previously tested protocol, including the fully remote nature, the prior feasibility and efficacy data could be suggestive that the treatment protocol is ready to be fully evaluated and tested.

2. Investigator(s):

Strengths

- The investigative team is impressive, with extensive experience in adolescent substance use, risk intervention, mobile health screening and intervention, primary care intervention, and qualitative data analyses.

Weaknesses

- None noted.

3. Innovation:

Strengths

- The age-specific approach to overcome barriers to treatment (e.g., using primary care for treatment entry, using apps and fully remove telehealth services) are notable strengths and innovative means of treatment implementation.
- Testing of fully remote oral fluid testing for cannabis abstinence is novel and could be broadly applicable to other research studies.
- The fully remote design, including components of telehealth, EMI app, and website, are modestly novel in full combination.

Weaknesses

- There are other effective telehealth apps for young adult CUD, including the already piloted app by this group. The MOMENT app, predecessor to MOMENT-V, has already been pilot tested on 70 participants and shown feasibility and efficacy.

4. Approach:

Strengths

- The EMI design incorporates feedback of prior pilot participants, increasing its engagement and effectiveness. EMI timing and frequency has strong supporting data to optimally engage participants over the duration of the intervention.
- There is a robust design for recruitment and assessment of outcomes, with clear benchmarks to delineate success of feasibility and efficacy aims. There are also clear markers of fidelity to the intervention.

Weaknesses

- It is unclear why some measures at follow-up gauge the full 3-month window (e.g., MPS), but not other components (e.g., TLFB).
- Exclusion criteria include “Medical or psychiatric condition that in the opinion of the PI would prevent safe participation in the study”. This is subject to significant bias, and exclusionary criteria should be justified and decided a priori.

5. Environment:

Strengths

- Environment is excellent both for the investigative team and the clinics involved in the study.

Weaknesses

- None noted.

Study Timeline:

Strengths

- Study timeline seems sufficient to accomplish launching and completing the study protocol, including analyses.

Weaknesses

- None noted.

Protections for Human Subjects:

Acceptable Risks and/or Adequate Protections

- Risks for participants are mior and include psychological discomfort during counseling, self-report survey completion, toxicological testing, or EMI intervention engagement. However, these risks are low, and mitigated through informed consent, working with well trained staff, access to contacting staff clinicians, a thorough data safety and montioring plan, and safety plan with clinical leadership. There is also risk of inadvertent loss of confidentiality. Standardized protocols and data protection protocols (including requiring participants to have passcodes on their smartphones) will mitigate loss of confidentiality risks.

Data and Safety Monitoring Plan (Applicable for Clinical Trials Only):

Acceptable

- o Weekly team meetings will monitoring all aspects of the study protocol, from recruitment througuh participant experience. A clear plan for addressing safety concerns, including during counseling sessions and including the participant's primary care team, is outlined. All monitoring will occur in real time. All data will be protected and stored on a secure drive, in HIPAA-compliant fashion.

Inclusion Plans:

- Sex/Gender: Distribution justified scientifically
- Race/Ethnicity: Distribution justified scientifically
- For NIH-Defined Phase III trials, Plans for valid design and analysis: Scientifically acceptable

- Inclusion/Exclusion Based on Age: Distribution justified scientifically
- This study focuses on young adults, which is appropriate given the prevalence of frequent cannabis use and CUD in this population. Further, there is a specific focus on understudied populations with cannabis use, though there are no specific recruitment crtieria by gender/sex or race or ethnicity.

Vertebrate Animals:

Not Applicable (No Vertebrate Animals)

Biohazards:

Not Applicable (No Biohazards)

Resubmission:

- The proposal is largely responsive to prior reviews, highlighting the innovative design components (including the expansion/revision of the intervention and inclusion of a website) and the additional study components (e.g., oral fluid testing; EMI with personalized messages). The resubmission appears to clarify the prior submission (e.g., recruitment strategies, EMI intervention design elements and rationalization), which is beneficial, but leaves some holes (e.g., not addressing comments regarding how preparation for an R01 will be determined, given the already extensive preliminary data contained within this proposal).

Applications from Foreign Organizations:

Not Applicable (No Foreign Organizations)

Select Agents:

Not Applicable (No Select Agents)

Resource Sharing Plans:

Acceptable

Authentication of Key Biological and/or Chemical Resources:

Not Applicable (No Relevant Resources)

Budget and Period of Support:

Recommend as Requested

CRITIQUE 2

Significance: 2
Investigator(s): 1
Innovation: 4

Approach: 3
Environment: 1

Overall Impact: This is a resubmitted R34 application that proposes a combination of a telehealth motivational enhancement therapy (MET) with mHealth ecological momentary intervention (EMI) to provide a brief intervention for 60 young adults aged 18-26 with cannabis use disorder who are recruited from primary care clinics. Follow up time points will be at 3 weeks, 3 months and 6 months. Aim 1 will determine 1) intervention feasibility (primary outcomes: intervention completion, EMI engagement, acceptability >80%), Aim 2) trial feasibility (primary outcomes: screening, eligibility, enrollment, retention), and Aim 3) preliminary efficacy to reduce 30-day cannabis use days and past-3-month problems from/negative consequences of use (self-report), each assessed at 3 and 6 months. An exploratory Aim will examine feasibility of both videorecorded and live oral fluid testing. This proposal has high public health significance in that there are few available interventions tailored towards young adults with cannabis use disorder. The proposal benefits from a strong environment and investigators with experience in treating young adults with substance use, EMI, and work in primary care settings. Despite the applicants response to initial reviewers justifying and updating some of the aspects of the intervention, the innovation of the proposal is still dampened as the components of the intervention including MET and brief intervention have been studied in this population before. Use of an external trained counselor to deliver the interventions (not necessarily embedded within the primary care clinic) limits generalizability. Weighing the strengths and weaknesses of the application, the potential impact of this study is likely moderate to high.

1. Significance:

Strengths

- Daily cannabis use continues to increase, particularly in young adults. At the same time, perceived risk of regular use is declining.
- There is a low rate of treatment receipt for cannabis use disorder in this population.
- Heavy cannabis exposure in this population increases risk of numerous adverse consequences including those on cognition, psychiatric disorders and high-risk behaviors.
- Fully remote interventions increase access and improve scalability.
- Employing the intervention in primary care increases reach.

Weaknesses

- None noted by this reviewer.

2. Investigator(s):

Strengths

- Complimentary experience across reviewers. PI Dr. Shrier is a board-certified Adolescent Medicine physician who provides expertise in mhealth, cannabis use, and ecological momentary interventions/assessments in youth.
- Co-I Dr. Harris brings expertise in developing and testing practical tools to promote screening, early identification, prevention, and treatment in the primary care setting of youth substance use and related risk behaviors.
- Co-I Dr. Katz-Wise will contribute expertise on mixed methods research in youth and young adults.

Weaknesses

- None noted by this reviewer.

3. Innovation:

Strengths

- Fully remote study procedures are novel and have not been widely tested in primary care settings.
- Investigating the feasibility of oral fluid self-testing for evaluating cannabis nonuse in a fully remote implementation of a CUD clinical trial is novel.

Weaknesses

- While the applicants addressed some of the concerns around innovation by highlighting new MET session activities, materials, format, and duration the concept of brief MET, the interventions are not novel in the study of CUD for young adults. This weakness (moderate) is mitigated by the use of mhealth and EMI.

4. Approach:

Strengths

- They have a plan to transfer the EMI to their own instance of a free mHealth application to increase dissemination potential and impact of the intervention.
- Engaging a diverse young adult advisory board is a helpful way to tailor recruitment strategies to improve diverse representation in research.
- Proposed intervention is based on extensive pilot research and testing.
- Use of enhanced usual care model is an excellent way to establish true efficacy of the intervention and prepare for a larger scale trial.

Weaknesses

- Generalizability becomes weakened when an external trained counselor (especially if a social worker or nurse practitioner) has to be brought into the intervention. They are also not really part of the clinic, so sustainability of these services in the future is unclear.
- It's unclear why the oral fluid testing is daily for four days (three days pre-visit and again during the visit) versus spaced over 1-2 weeks to more accurately capture use across the duration of the intervention.
- Definition of "problematic cannabis use" for the Young Adult Advisory Board is unclear.

5. Environment:

Strengths

- Boston Children's Hospital is well suited to support this study.
- Primary care clinics are an ideal study setting for the intervention. Boston Children's Hospital Adolescent/Young Adult Medical Practice and Boston Children's Primary Care at Martha Eliot Adolescent and Young Adult Clinic appear to have the number of necessary participants from which to recruit.

Weaknesses

- None noted by this reviewer.

Study Timeline:

Strengths

- Timeline is appropriate as indicated.

Weaknesses

- None noted by this reviewer.

Protections for Human Subjects:

Acceptable Risks and/or Adequate Protections

Data and Safety Monitoring Plan (Applicable for Clinical Trials Only):

Acceptable

- Use of DSMP: PI Oversight, a Participant Safety plan, Adverse Event reporting, and Data Management and Monitoring.

Inclusion Plans:

- Sex/Gender: Distribution justified scientifically
- Race/Ethnicity: Distribution justified scientifically
- For NIH-Defined Phase III trials, Plans for valid design and analysis: Not applicable
- Inclusion/Exclusion Based on Age: Distribution justified scientifically

Vertebrate Animals:

Not Applicable (No Vertebrate Animals)

Biohazards:

Not Applicable (No Biohazards)

Resubmission:

- Previous reviewers noted a lack of innovation in the intervention. The applicants clarified that while they have used some of the components of the intervention in the past, the combination is novel including new activities, materials, format, and duration. This appears to only partly justify innovation of the content. The applicants also incresed the linkage between EMI and the MET intervention. Oral fluid testing is now used to verify claims of reduction/abstinence for the primary analyses. They also addressed comments regarding trial eligibility, YAAB eligibility, EMI prompt scheduling and message timing, fidelity monitoring, and payment.

Applications from Foreign Organizations:

Not Applicable (No Foreign Organizations)

Select Agents:

Not Applicable (No Select Agents)

Resource Sharing Plans:

Acceptable

Authentication of Key Biological and/or Chemical Resources:

Not Applicable (No Relevant Resources)

Budget and Period of Support:

Recommend as Requested

CRITIQUE 3

Significance: 1
Investigator(s): 3
Innovation: 3
Approach: 6
Environment: 1

Overall Impact: The proposed project involves evaluating a fully remote intervention for young adult cannabis use that combines telehealth therapy with an mHealth ecological momentary intervention (EMI). The investigators will determine intervention feasibility, trial feasibility, and preliminary efficacy of the intervention as compared to enhanced usual care. The intervention is backed by promising preliminary work that demonstrates engagement with the MOMENT intervention and a reduction in cannabis desire. A score-driving concern, however, is that critical information about the mHealth application that is central to the intervention is missing, such as the user interface and intervention workflow. There are also concerns that the EMI responses may be missed due to (1) certain contextual factors the participants may be in such as social settings and (2) the reliance on app surveys that can be easily missed compared to text messages, although preliminary work demonstrated that participants often did read the messages. Overall, however, the strengths of this proposal outweigh the weaknesses, leading to a medium-to-high impact proposal.

1. Significance:

Strengths

- mHealth interventions have the potential to reduce cannabis use in young adults if designed effectively.

Weaknesses

- None noted.

2. Investigator(s):

Strengths

- PI has a track record for developing technology-based substance use interventions and EMAs, including for cannabis use.
- Other investigators and team members bring complementary expertise in adolescent medicine, biostatistics, qualitative data analysis, study design and trial execution for substance use brief interventions, and substance use.

Weaknesses

- There is no investigator or team member with expertise in mHealth. This can limit the ability to make app modifications that the Young Adult Advisory Board may suggest. This limits the usefulness of having the YAAB.

3. Innovation:

Strengths

- The use of tailored messaging and EMIs for cannabis is innovative.

Weaknesses

- EMAs are commonly used in substance use research.

4. Approach:

Strengths

- Extensive preliminary studies provide support for the acceptability and feasibility of MOMENT and identified areas for improvement that are addressed in the proposed research. The studies also demonstrated that participants read the messages and otherwise engaged with the app.

Weaknesses

- Crucial details about the MOMENT-V mHealth app are missing, particularly in regard to the user interface and interaction flow of the mHealth app. No screenshots are provided.
- It is unlikely that someone will respond to an EMI in certain trigger settings, such as being at a friend’s house, due to the social pressures of being in that environment. This limits the ability for a just-in-time intervention.
- A reliance on app surveys leads to the risk of non-response, as opening an app can be more burdensome than responding to a text and app notifications are easier to miss due to notification fatigue.
- There are no details about the message assignment algorithm provided.
- There is no plan to iterate on the EMI application based on feedback.

5. Environment:

Strengths

- BCH provides all resources needed for running the proposed trial.

Weaknesses

- None noted.

Study Timeline:

Strengths

- The proposed timeline is feasible.

Weaknesses

- None noted.

Protections for Human Subjects:

Acceptable Risks and/or Adequate Protections

- Adequate security and privacy protections are in place.

Data and Safety Monitoring Plan (Applicable for Clinical Trials Only):

Acceptable

- Frequent monitoring by the study team will occur.

Inclusion Plans:

- Sex/Gender: Distribution justified scientifically
- Race/Ethnicity: Distribution justified scientifically
- For NIH-Defined Phase III trials, Plans for valid design and analysis: Not applicable
- Inclusion/Exclusion Based on Age: Distribution justified scientifically
- All distributions are justified.

Vertebrate Animals:

Not Applicable (No Vertebrate Animals)

Biohazards:

Not Applicable (No Biohazards)

Resubmission:

- Previous critiques are adequtely addressed.

Applications from Foreign Organizations:

Not Applicable (No Foreign Organizations)

Select Agents:

Not Applicable (No Select Agents)

Resource Sharing Plans:

Acceptable

Authentication of Key Biological and/or Chemical Resources:

Not Applicable (No Relevant Resources)

Budget and Period of Support:

Recommend as Requested

THE FOLLOWING SECTIONS WERE PREPARED BY THE SCIENTIFIC REVIEW OFFICER TO SUMMARIZE THE OUTCOME OF DISCUSSIONS OF THE REVIEW COMMITTEE, OR REVIEWERS' WRITTEN CRITIQUES, ON THE FOLLOWING ISSUES:

PROTECTION OF HUMAN SUBJECTS: ACCEPTABLE

INCLUSION OF WOMEN PLAN: ACCEPTABLE

INCLUSION OF MINORITIES PLAN: ACCEPTABLE

INCLUSION ACROSS THE LIFESPAN: ACCEPTABLE

COMMITTEE BUDGET RECOMMENDATIONS: The budget was recommended as requested.

Footnotes for 1R34DA060500-01A1; PI Name: SHRIER, LYDIA A.

NIH has modified its policy regarding the receipt of resubmissions (amended applications).See Guide Notice NOT-OD-18-197 at https://grants.nih.gov/grants/guide/notice-files/NOT-OD-18-197.html. The impact/priority score is calculated after discussion of an application by averaging the overall scores (1-9) given by all voting reviewers on the committee and multiplying by 10. The criterion scores are submitted prior to the meeting by the individual reviewers assigned to an application, and are not discussed specifically at the review meeting or calculated into the overall impact score. Some applications also receive a percentile ranking. For details on the review process, see http://grants.nih.gov/grants/peer_review_process.htm#scoring.

MEETING ROSTER

Interventions to Prevent and Treat Addictions Study Section
Risk, Prevention and Health Behavior Integrated Review Group
CENTER FOR SCIENTIFIC REVIEW
IPTA
06/13/2024 - 06/14/2024

Notice of NIH Policy to All Applicants: Meeting rosters are provided for information purposes only. Applicant investigators and institutional officials must not communicate directly with study section members about an application before or after the review. Failure to observe this policy will create a serious breach of integrity in the peer review process, and may lead to actions outlined in NOT-OD-22-044 at https://grants.nih.gov/grants/guide/notice-files/NOT-OD-22-044.html, including removal of the application from immediate review.

CHAIRPERSON(S)

PIPER, MEGAN E, PHD
PROFESSOR
DEPARTMENT OF MEDICINE
UNIVERSITY OF WISCONSIN-MADISON
MADISON, WI 53711

MEMBERS

AUDRAIN-MCGOVERN, JANET, PHD
PROFESSOR AND DIRECTOR
DEPARTMENT OF PSYCHIATRY
PERELMAN SCHOOL OF MEDICINE
UNIVERSITY OF PENNSYLVANIA
PHILADELPHIA, PA 19104

BINSWANGER, INGRID A, MD
SENIOR CLINICIAN INVESTIGATOR
INSTITUTE FOR HEALTH RESEARCH
KAISER PERMANENTE
AURORA, CO 80014

BREWER, JUDSON A, PHD, MD *
PROFESSOR
DEPARTMENT OF BEHAVIORAL AND SOCIAL SCIENCES
DEPARTMENT OF PSYCHIATRY
BROWN UNIVERSITY
PROVIDENCE, RI 02903

CLAUS, ERIC D, PHD
ASSOCIATE PROFESSOR
DEPARTMENT OF BIOBEHAVIORAL HEALTH
THE PENNSYLVANIA STATE UNIVERSITY
UNIVERSITY PARK, PA 16802

COCHRAN, GERALD T, PHD *
PROFESSOR
DEPARTMENT OF INTERNAL MEDICINE
DIVISION OF EPIDEMIOLOGY
UNIVERSITY OF UTAH
SALT LAKE CITY, UT 84132

DIBELLO, ANGELO MICHAEL, PHD *
ASSISTANT PROFESSOR
DEPARTMENT OF GRADUATE SCHOOL OF APPLIED AND PROFESSIONAL PSYCHOLOGY
RUTGERS UNIVERSITY
PISCATAWAY, NJ 08854

EBRAHIMI KALAN, MOHAMMAD, PHD *
ASSISTANT PROFESSOR
SCHOOL OF HEALTH PROFESSION
EASTERN VIRGINIA MEDICAL SCHOOL,
NORFOLK, VA 23507

ERTIN, EMRE, PHD *
ASSOCIATE PROFESSOR
DEPARTMENT OF ELECTRICAL AND COMPUTER ENGINEERING
COLLEGE OF ENGINEERING
THE OHIO STATE UNIVERSITY
COLUMBUS, OH 43210

FRIDBERG, DANIEL J, PHD *
ASSOCIATE PROFESSOR
DEPARTMENT OF PSYCHIATRY AND BEHAVIORAL NEUROSCIENCE
THE UNIVERSITY OF CHICAGO
CHICAGO, IL 60637

HAGLE, HOLLY, PHD *
ASSOCIATE RESEARCH PROFESSOR
MISSOURI CENTER FOR ADDICTION RESEARCH AND ENGAGEMENT
COLLEGE OF ARTS AND SCIENCE
UNIVERSITY OF MISSOURI
KANSAS CITY, MO 64110

KLEMPERER, ELIAS MUSHABAC, PHD *
ASSISTANT PROFESSOR
DEPARTMENT OF PSYCHIATRY
LARNER COLLEGE OF MEDICINE
UNIVERSITY OF VERMONT
BURLINGTON, VT 05405

KMIEC, JULIE, DO *
ASSOCIATE PROFESSOR
DEPARTMENT OF PSYCHIATRY
UNIVERSITY OF PITTSBURGH
PITTSBURGH, PA 15213

KONG, GRACE, PHD
ASSOCIATE PROFESSOR
DEPARTMENT OF PSYCHIATRY
YALE UNIVERSITY SCHOOL OF MEDICINE
NEW HAVEN, CT 06519

KOWALCHUK, ALICIA ANN, DO *
ASSOCIATE PROFESSOR
BAYLOR COLLEGE OF MEDICINE
HOUSTON, TX 77030

LEDGERWOOD, DAVID M, PHD
PROFESSOR
DEPARTMENT OF PSYCHIATRY
AND BEHAVIORAL NEUROSCIENCES
SCHOOL OF MEDICINE
WAYNE STATE UNIVERSITY
DETROIT, MI 48201

LEE, DUSTIN CLARK, PHD *
ASSISTANT PROFESSOR
DEPARTMENT OF PSYCHIATRY AND BEHAVIORAL
SCIENCES
THE JOHNS HOPKINS SCHOOL OF MEDICINE
BALTIMORE, MD 21224

LOPEZ-CASTRO, TERESA, PHD *
ASSOCIATE PROFESSOR
DEPARTMENT OF PSYCHOLOGY
CITY COLLEGE OF NEW YORK
NEW YORK, NY 10031

MARK, KATRINA, MD *
ASSOCIATE PROFESSOR
DEPARTMENT OF OBSTETRICS, GYNECOLOGY
AND REPRODUCTIVE SCIENCES
UNIVERSITY OF MARYLAND SCHOOL OF MEDICINE
BALTIMORE, MD 21201

MCHUGH, REBECCA KATHRYN, PHD
ASSOCIATE PROFESSOR
DEPARTMENT OF PSYCHIATRY
HARVARD MEDICAL SCHOOL
BELMONT, MA 02478

MCNEELY, JENNIFER, MD
ASSOCIATE PROFESSOR
DEPARTMENTS OF POPULATION HEALTH AND MEDICINE
GROSSMAN SCHOOL OF MEDICINE
NEW YORK UNIVERSITY
NEW YORK, NY 10016

MCPHERSON, STERLING M, PHD
DIRECTOR AND PROFESSOR
PROGRAM OF EXCELLENCE IN ADDICTIONS RESEARCH
DEPARTMENT OF COMMUNITY AND BEHAVIORAL HEALTH
ELSON S. FLOYD COLLEGE OF MEDICINE
WASHINGTON STATE UNIVERSITY
SPOKANE, WA 99210

MONTGOMERY, LATRICE, PHD
DIRECTOR OF CLINICAL RESEARCH AT RIA HEALTH,
ADJUNCT ASSOCIATE PROFESSOR
DEPARTMENT OF PSYCHIATRY AND
BEHAVIORAL NEUROSCIENCE
COLLEGE OF MEDICINE
UNIVERSITY OF CINCINNATI
CINCINNATI, OH 45229

OSTACHER, MICHAEL JOSHUA, MD, MPH *
PROFESSOR
DEPARTMENT OF PSYCHIATRY AND BEHAVIORAL
SCIENCES
STANFORD UNIVERSITY
PALO ALTO, CA 94304

RIDENOUR, TY A, PHD *
RESEARCH PUBLIC HEALTH ANALYST
CENTER FOR SOCIAL DETERMINANTS,
RISK FACTORS, AND PREVENTION SCIENCE
RESEARCH TRIANGLE INSTITUTE
RESEARCH TRIANGLE PARK, NC 27709

SADASIVAM, RAJANI, PHD
PROFESSOR
DIVISION OF HEALTH AND IMPLEMENTATION SCIENCE
DEPARTMENT OF POPULATION AND QUANTITATIVE
HEALTH SCIENCES
UNIVERSITY OF MASSACHUSETTS MEDICAL SCHOOL
WORCESTER, MA 01605

SCHWEBEL, FRANK, PHD *
RESEARCH ASSISTANT PROFESSOR
CENTER ON ALCOHOL, SUBSTANCE USE, AND ADDICTIONS
THE UNIVERSITY OF NEW MEXICO
ALBUQUERQUE, NM 87123

SCOTT, KELLI, PHD *
ASSISTANT PROFESSOR
DEPARTMENT OF MEDICAL SOCIAL SCIENCES
FEINBERG SCHOOL OF MEDICINE
NORTHWESTERN UNIVERSITY
CHICAGO, IL 60613

SPEARS, CLAIRE ADAMS, PHD
ASSOCIATE PROFESSOR
DEPARTMENT OF HEALTH POLICY & BEHAVIORAL SCIENCES
SCHOOL OF PUBLIC HEALTH
GEORGIA STATE UNIVERSITY
ATLANTA, GA 30303

SPILLANE, NICHEA SOLOMON, PHD
ASSOCIATE PROFESSOR
DEPARTMENT OF PSYCHOLOGY
COLLEGE OF HEALTH SCIENCES
UNIVERSITY OF RHODE ISLAND
KINGSTON, RI 02881

STEVENS-WATKINS, DANELLE J, PHD
PROFESSOR
DEPARTMENT OF EDUCATIONAL, SCHOOL, AND COUNSELING PSYCHOLOGY
COLLEGE OF EDUCATION
UNIVERSITY OF KENTUCKY
LEXINGTON, KY 40506

STORMSHAK, ELIZABETH A, PHD
PROFESSOR
COLLEGE OF EDUCATION
PREVENTION SCIENCE INSTITUTE
UNIVERSITY OF OREGON
EUGENE, OR 97403

WADE, NATASHA E, PHD *
ASSISTANT PROFESSOR
DEPARTMENT OF PSYCHIATRY
UNIVERSITY OF CALIFORNIA, SAN DIEGO
LA JOLLA, CA 92093

WASHINGTON, PETER, PHD *
ASSISTANT PROFESSOR
INFORMATION & COMPUTER SCIENCES DEPARTMENT
UNIVERSITY OF HAWAII AT MĀNOA
HONOLULU, HI 96822

WELSH, JUSTINE WITTENAUER, MD *
ASSOCIATE PROFESSOR
DEPARTMENT OF PSYCHIATRY AND BEHAVIORAL SCIENCES
EMORY UNIVERSITY SCHOOL OF MEDICINE
ATLANTA, GA 30307

YI, RICHARD, PHD
PROFESSOR AND DIRECTOR
COFRIN LOGAN CENTER FOR ADDICTION RESEARCH AND TREATMENT
DEPARTMENT OF PSYCHOLOGY
UNIVERSITY OF KANSAS
LAWRENCE, KS 66045

SCIENTIFIC REVIEW OFFICER

ZANDBERG, IZABELLA, PHD
SCIENTIFIC REVIEW OFFICER
CENTER FOR SCIENTIFIC REVIEW
BETHESDA, MD 20892

EXTRAMURAL SUPPORT ASSISTANT

AMARE, MERON ERMIAS
LEAD EXTRAMURAL SUPPORT ASSISTANT
CENTER FOR SCIENTIFIC REVIEW
NATIONAL INSTITUTES OF HEALTH
BETHESDA, MD 20892

* Temporary Member. For grant applications, temporary members may participate in the entire meeting or may review only selected applications as needed.

Consultants are required to absent themselves from the room during the review of any application if their presence would constitute or appear to constitute a conflict of interest.
